# Comparison of Production and Fluorescence Characteristics of Phycoerythrin from Three Strains of *Porphyridium*

**DOI:** 10.3390/foods11142069

**Published:** 2022-07-12

**Authors:** Chulin Li, Houbo Wu, Wenzhou Xiang, Hualian Wu, Na Wang, Jiayi Wu, Tao Li

**Affiliations:** 1 CAS Key Laboratory of Tropical Marine Bio-resources and Ecology, Guangdong Key Laboratory of Marine Materia Medica, Institution of South China Sea Ecology and Environmental Engineering, South China Sea Institute of Oceanology, Chinese Academy of Sciences, Guangzhou 510301, China; lchlxpy@126.com (C.L.); wuhoubo@scsio.ac.cn (H.W.); xwz@scsio.ac.cn (W.X.); hlwu@scsio.ac.cn (H.W.); nawang@scsio.ac.cn (N.W.); kayeewu@scsio.ac.cn (J.W.); 2 Southern Marine Science and Engineering Guangdong Laboratory (Guangzhou), Guangzhou 511458, China

**Keywords:** *Porphyridium*, phycoerythrin, fluorescence characteristics, RNA-seq

## Abstract

Phycoerythrin, a special photosynthetic pigment, is widely used as fluorescent dye and has lots of underlying beneficial effects on health. A marine red microalga *Porphyridium* is considered as the potential feedstock for phycoerythrin production. However, the phycoerythrin-related properties of *Porphyridium* have not been systematically evaluated, especially between the species of *P. cruentum* and *P. purpureum*. The present study aimed to evaluate the production and fluorescence characteristics of phycoerythrin of three strains of *Porphyridium*. The results showed that *P. purpureum* SCS-02 presented the highest biomass, phycoerythrin content and yield were 6.43 g L^−1^, 9.18% DW and 0.288 g L^−1^, respectively. There was no significant difference between *P. purpureum* and *P. cruentum* in α and β subunits amino acid sequences of phycoerythrin and in fluorescence characteristics. The high gene expression level of the key enzymes in phycoerythrobilin synthesis (porphobilinogen synthase and oxygen-dependent coproporphyrinogen-III oxidase) could be related to the high phycoerythrin content of *Porphyridium*. Based on systematic evaluation, *P. purpureum* SCS-02 was selected due to its high biomass and phycoerythrin yield. *P. purpureum* and *P. cruentum* were highly similar in the phylogenetic tree, as well as in fluorescence characteristics; therefore, it was speculated that they might be the same *Porphyridium* species.

## 1. Introduction

Phycoerythrin, a photosynthetic pigment in Rhodophyta, Cyanobacteria and Cryptophytes, producing orange fluorescence spontaneously with high fluorescence quantum yield (up to 0.98), is a rare macromolecular pigment protein complex in nature. Currently, it is widely used as fluorescent dye. Additionally, the potential functions of phycoerythrin were reported, including modulating the gut microbiota [1], inducing apoptosis of cancer cells [2], regulating immunity [3,4], anti-allergy and anti-aging [5]. Thus, phycoerythrin is considered as a natural high-value product in future.

*Porphyridium*, belonging to Bangiophyceae, Porophyridiales and Porophyridiaceae, is a single-celled red algae often found in seawater and moist soil. Moreover, *Porphyridium* contains a special star-shaped chloroplast with phycoerythrin inside. The phycoerythrin content of *Porphyridium* is about 5–10% DW (dry weight), which is significantly higher than that of other phycoerythrin-containing algal species, such as Cyanobacteria of *Anabaena* sp. (1.23% DW) [6] and *Pseudanabaena* sp. (4.17% DW) [7,8], Rhodophyta of *Gracilaria gracilis* (0.09–0.12% DW) [9,10,11], *Neopyropia haitanensis* (0.25% DW) [12] and *Neopyropia yezoensis* (0.80% DW) [13]. Therefore, *Porphyridium* is an ideal algal strain for phycoerythrin production.

Microalgae derived from different natural environments might differ in growth, active substance contents and environmental adaptability [14,15]. Therefore, based on different applications, the screening and evaluating of microalgal strains should be conducted. Zhao et al. (2018) obtained a strain of *Chlorella zofingiensis* with high denitrification and dephosphorization efficiency, which could be applied in waste treatment [16]. Rodolfi et al. (2009) screened *Nannochloropsis* sp. F&M-M24 with 60% DW content of oil from 30 strains of freshwater and marine microalgae and predicted that 30 tons of oil per hectare could be obtained in sunny tropical areas [17]. Among the strains reported in previous studies, the contents of phycoerythrin were quite different. The phycoerythrin content of *Porphyridium purpureum* SCS-02 cultivated at 17.6 mM NaNO_3_ was 8.18% DW [18]. Gudvilovich et al. (2021) showed that the phycoerythrin content of *P. purpureum* IBSS-70 could reach up to 5.50% DW [19]. The phycoerythrin content of *P. purpureum* CCMP1328 reported by Li et al. (2020) was only 2.00% DW [20]. In the present study, three *Porphyridium* strains, including *Porphyridium cruentum* CCALA 415 (purchased), *P. purpureum* SCS-02 (isolated by our laboratory) and *P. purpureum* FACHB 806 (purchased), were used as experimental materials. *P. cruentum* and *P. purpureum* are two of the most studied species of *Porphyridium*. However, few systematic comparisons of them have been conducted, especially for the phycoerythrin-related properties. Importantly, there has been a taxonomic confusion between *P. cruentum* and *P. purpureum* that some scholars consider them to be the same species [21,22,23,24], while others treat them as two different species [25,26,27].

In this study, the morphological characteristics, 18s rDNA and ITS sequences, growth, the content and fluorescence characteristics of phycoerythrin and the key genes expression level in phycoerythrin synthesis were investigated to compare the differences between three strains of *Porphyridium* (*P. cruentum* CCALA-415, *P. purpureum* SCS-02 and *P. purpureum* FACHB-806). A potential strain of *Porphyridium* with the fast growth rates, high phycoerythrin yield and excellent fluorescence characteristics will be expected to be selected for the large-scale production of phycoerythrin in future.

## 2. Materials and Methods

### 2.1. Strains and Culture Conditions

Three strains of *Porphyridium* from different algae collection centers were used as experimental materials. *P. cruentum* CCALA 415, isolated in 1935, was purchased from the Culture Collection of Autotrophic Organisms (https://ccala.butbn.cas.cz/en (accessed on 10 January 2022)) in the Czech Republic. The Culture Collection of Autotrophic Organisms cannot provide information on the original collection site. *P. purpureum* FACHB 806, originally isolated by the Ocean University of China, was purchased from the Freshwater Algae Culture Collection of the Institute of Hydrobiology (http://algae.ihb.ac.cn/ (accessed on 10 January 2022)) in China. *P. purpureum* SCS-02 was isolated by our laboratory from the seawater samples (from the South China Sea) [18].

All three strains of *Porphyridium* were cultured in the modified ASW medium [18]. The culture temperature was maintained at 25 ± 1 °C. The single-sided T8 fluorescent lamp (Philips, Shanghai, China) with a light intensity of 130 μmol·photons m^−2^·s^−1^ was used for 24 h:0 h (light:dark). The compressed air with 1% CO_2_ (air: CO_2_, 99: 1) was continuously bubbled in photobioreactor to supply carbon.

### 2.2. Experimental Design

Three strains of *Porphyridium* cultured to the logarithmic growth stage were reinoculated into 300 mL columnar photobioreactors (Φ3 cm × 60 cm) with an initial density of OD_750_ = 0.40 ± 0.02. Three biological replicates were set for each treatment. On days 0, 2, 4, 6, 8, 10, 12 and 14, samples were taken to determine biomass concentration and phycoerythrin content. On day 8, the algal cells were collected for total RNA extraction and transcriptome sequencing. The wet biomass on day 14 was used to extract and purify phycoerythrin to evaluate the fluorescence characteristics.

### 2.3. Morphological Observation

Cell morphology was observed by using a BX53 light microscope (Olympus, Tokyo, Japan).

### 2.4. Construction of Phylogenetic Tree

DNA was extracted by using EZNA Plant DNA Kit (Omega Bio-Tek, Norcross, GA, USA). Amplification was performed using 2720 PCR Thermal Cycler (Applied Biosystems, Foster City, CA, USA) with a reaction system of 50.0 μL (2.0 μL forward and reverse primers (Table 1), 2.5 μL DMSO, 25.0 μL Premix Taq DNA polymerase (version 2.0 plus dye) (Takara, Tokyo, Japan), 16.5 μL ddH_2_O, 2.0 μL DNA sample). PCR amplification denaturation, annealing, and denaturation temperatures were 94 °C, 52 °C, and 72 °C, respectively, with 36 cycles. The amplified DNA products were sequenced by Sangon Biotech Co., Ltd. (Shanghai, China).

The 18s rDNA sequences and internal transcribed spacer (ITS) regions were aligned at the NCBI website. Multiple sequence alignment (based on MUSCLE algorithm) was performed using MEGA-X software [28], and then the 18s rDNA and ITS phylogenetic trees were constructed according to the Neighbor-Joining method and the Minimum Evolution method, respectively, with bootstrap analysis of 1000 replicates.

The base sequences of the α and β subunits of phycoerythrin obtained from sequencing were translated into amino acid sequences and aligned at the NCBI website. Amino acid sequence analysis was performed using BioEdit version 7.2 software.

### 2.5. Growth Measurement

A 10 mL culture was filtered through a 0.45 μm pre-weighed filter membrane (Tianjin jinteng Experimental Equipment Co., Ltd., Tianjin, China). The filters were dried at 80 °C for 12 h. The biomass concentration was calculated according to the following equation:

DW = (M_1_ − M_0_)/V (1)

where DW is the biomass concentration (g L^−1^). M_0_ is the unloaded filter membrane mass (g). M_1_ is the algae-loaded filter membrane mass (g). V is the sampling volume (L).

### 2.6. Determination of Phycobiliproteins Content

The wet biomass from day 0 to day 14 were collected by centrifugation at 8000 rpm for 10 min. The wet biomass was added to 5 mL of 20 mM Tris-HCl buffer (pH 8.0) and placed alternately at −20 °C and 4 °C for 24 h for repeated freeze-thawing. Then, the alga biomass was removed by centrifugation at 8000 rpm for 10 min. The absorbance of the extract at 565 nm, 620 nm and 650 nm were measured by TU-1810 UV-Visible spectrophotometer (Persee Instrument Co., Ltd., Beijing, China). The phycobiliproteins content were calculated by the following equations [29]:

C_R-PC_ = (OD_620_ − 0.7 × OD_650_)/7.38 (2)

C_APC_ = (OD_650_ − 0.19 × OD_620_)/5.65 (3)

C_B-PE_ = (OD_565_ − 2.8 × C_R-PC_ − 1.34 × C_APC_)/12.7 (4)

R-PC (% DW) = (C_R-PC_ × 5)/(DW × V) × 100% (5)

B-PE (% DW) = (C_B-PE_ × 5)/(DW × V) × 100% (6)

The resulting B-PE (% DW) was used to calculate the yield of B-phycoerythrin by the following equation:

B-PE (g L^−1^) = B-PE (% DW) × DW (7)

where C_R-PC_, C_APC_ and C_B-PE_ are the contents of R-phycocyanin, allophycocyanin and B-phycoerythrin in the extracts, respectively (mg mL^−1^). OD_565_, OD_620_ and OD_650_ are the optical density values at 565 nm, 620 nm and 650 nm, respectively. DW is the biomass concentration (g L^−1^). V is the volume of the collected algal solution (L).

### 2.7. Purification of Phycoerythrin

At the end of the cultivation, 600 mL of the culture was centrifuged to collect the algal sludges. The sludges were placed in 20 mL of 20 mM Tris-HCl buffer (pH 8.0). Repeated freezing and thawing 3−4 times to extract took place until no red in algae cells. The extract was filtered using 0.45 μm microporous filter membrane (Tianjin jinteng Experimental Equipment Co., Ltd., Tianjin, China) and then loaded onto a HiPrep DEAE FF 16/10 column (GE Healthcare, Chicago, IL, USA) to elute with a gradient of 0–1.0 M NaCl. The purified eluent was concentrated using ultrafiltration centrifuge tubes (10,000 MWCO) (Millipore Corporation, Bedford, MA, USA) and then loaded onto a Superdex 200 Increase 10/300 GL column (GE Healthcare, Chicago, IL, USA) to further purify with elution buffer (200 mM NaCl and 20 mM Tris-HCl (pH 8.0)). The absorbance of phycoerythrin at 280 nm and 545 nm were measured using TU-1810 UV-Visible spectrophotometer. The optical density at the wavelength of 280 nm and 545 nm were used to represent the concentration of crude total protein and B-phycoerythrin, respectively [30,31,32,33]. The purity of B-phycoerythrin was expressed by the following equation:

The purity of B-phycoerythrin = OD_545_/OD_280_ (8)

where B-PE is B-phycoerythrin. OD_280_ and OD_545_ are the optical density values at 280 nm and 545 nm, respectively.

### 2.8. Determination of Fluorescence Characteristics of Phycoerythrin

The OD_545_ of the purified phycoerythrin solution was adjusted to 0.500 ± 0.010. And the concentration of the purified phycoerythrin in the three strains of *Porphyridium* were about 0.04 mg mL^−1^. The spectra at 350–800 nm were determined by using UV-visible spectrophotometer (Persee Instrument Co., Ltd., Beijing, China). Fluorescence emission spectra were determined by using an F97 Pro fluorescence spectrophotometer (Lengguang Technology, Shanghai, China) with excitation wavelength at 545 nm. The 200−800 nm fluorescence excitation spectra were determined at the emission wavelength of 578 nm. Then, the OD_545_ of the purified phycoerythrin solution was adjusted to 0.1, 0.2, 0.3, 0.4 and 0.5, respectively, and the fluorescence emission spectra were determined at the excitation wavelength of 545 nm. The above operations were repeated twice, and all operations were conducted in the dark.

### 2.9. RNA-seq

#### 2.9.1. Total RNA Extraction, cDNA Library Construction and Sequencing

The algae cells were collected by centrifugation at 8000 rpm for 10 min. Then liquid nitrogen was quickly added for grinding. Total RNA was extracted using the RNA extraction kit. Its concentration, purity and integrity were measured by using NanoDrop 2000 ultra-micro spectrophotometer (Thermo Scientific, Waltham, MA, USA) and Agilent 2100 Bioanalyzer (Agilent Technologies, Santa Clara, CA, USA), respectively. Subsequently, mRNA was enriched using Oligo (dT) magnetic beads, and broken into fragments of about 300 bp by the action of metal ions. The cDNA library was constructed using 6 base pair (bp) random primers and reverse transcriptase with mRNA as the template, and then the library fragments were enriched by PCR amplification. Constructed cDNA library underwent a quality inspection by using Agilent 2100 Bioanalyzer. Finally, the cDNA library was paired-end sequenced based on the Illumina sequencing platform.

#### 2.9.2. Data Analysis

After sequencing, raw reads were filtered (including removing adaptors and low-quality sequences) by Cutadapt software. The quality assessment was performed by calculating Q_20_, Q_30_ and GC contents by FastQC software. The clean reads (filtered high quality sequences) were aligned to the reference genome (*P. purpureum* CCMP1328 genome (GCA_008690995.1) and *P. purpureum* CCMP1328 plastid complete genome (MF401423.1)) by HISAT2 software. Subsequently, fragments per kilobase of transcript per million fragments mapped (FPKM) was applied to calculate the expression levels of unigenes. Differentially expressed genes were screened by DESeq software (*p*-value < 0.05 and |log_2_FoldChange| > 1).

### 2.10. Statistical Analysis

The means and standard deviations shown in all figures and tables were obtained from three biological replicates and two technical replicates. One-way ANOVA was performed using SPSS18.0 statistical software (SPSS Inc., Chicago, IL, USA). The least significant difference (LSD) at α = 0.05 was used to indicate that the treatment groups were significantly different at the mean level.

## 3. Results

### 3.1. Molecular Identification and Cell Morphology

**Figure 1:**
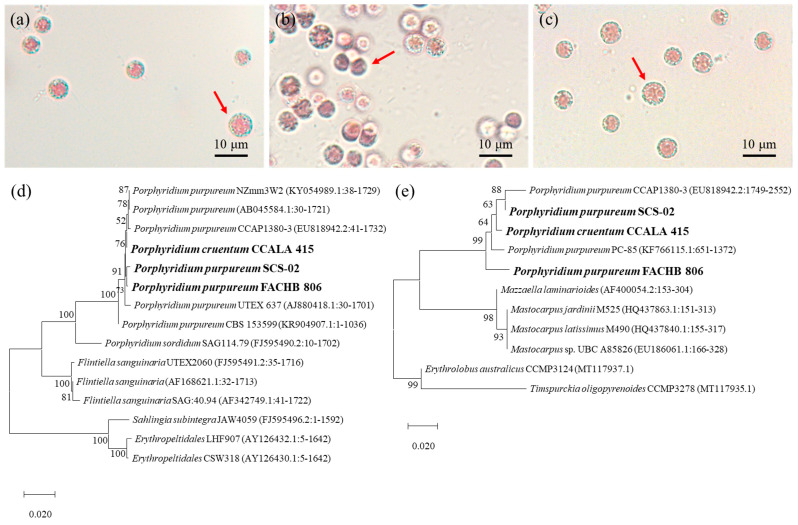
Cell morphology of *P. cruentum* CCALA-415 (**a**), *P. purpureum* SCS-02 (**b**) and *P. purpureum* FACHB-806 (**c**). Phylogenetic tree based on 18s rDNA gene sequence (**d**) and internal transcribed spacer regions (**e**) of the three strains of *Porphyridium*.

As shown in Figure 1a–c, CCALA-415, SCS-02 and FACHB-806 were similar in cell morphology and cell size. The cells were spheroidal in shape, 5–7 μm in diameter, and wrapped with a transparent sheath outside the cell. Notably, SCS-02 showed a darker red color compared to CCALA-415 and FACHB-806. The 18s rDNA phylogenetic trees of CCALA-415, SCS-02 and FACHB-806 showed that three strains of *Porphyridium* had high homology (Figure 1d). Among them, the homology between “CCALA-415 vs. SCS-02”, “CCALA-415 vs. FACHB-806” and “SCS-02 vs. FACHB-806” were 99.82%, 100% and 99.82%, respectively. In addition, BLAST comparison revealed that SCS-02 had high homology with *P. purpureum* CCAP1380-3 (99.65%), *P. purpureum* NZmm3W2 (99.59%), *P. purpureum* UTEX 637 (99.16%) and *P. purpureum* CBS 153599 (96.93%). The ITS regions of three strains also showed high homology (Figure 1e). Moreover, higher homology was found between “SCS-02 vs. CCALA-415” (96.95%) compared to “CCALA-415 vs. FACHB-806 (96.69%)” and “SCS-02 vs. FACHB-806” (96.21%).

### 3.2. Growth Characteristic

**Figure 2:**
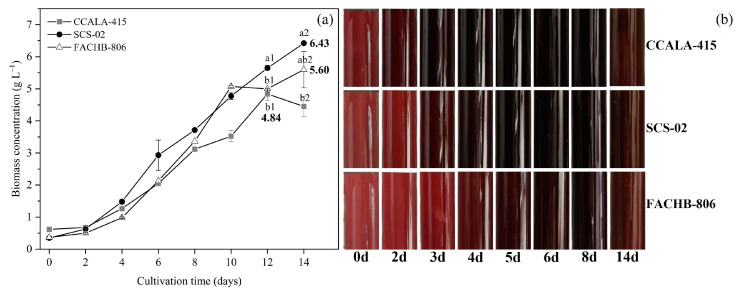
Growth characteristics of *Porphyridium* *cruentum* CCALA-415, *Porphyridium* *purpureum* SCS-02 and *Porphyridium* *purpureum* FACHB-806. (**a**) Biomass concentration from day 0 to day 14; (**b**) The color change of the culture in the photobioreactors as a function of time. The values shown in Figure 2a are the averages of two biological replicates and three technical replicates ± standard deviation. Different letters denote significant differences among the values of biomass concentration in the three strains of *Porphyridium* (a1–c1: day 12; a2–c2: day 14) (level of significance, *p* < 0.05).

As shown in Figure 2a, the growth trends of the three strains of *Porphyridium* were different. From day 0 to day 4, there was no significant difference in the biomass concentration. From day 4 to day 10, SCS-02 had a higher biomass concentration compared to CCALA-415 and FACHB-806. After day 10, the growth rates of CCALA-415 and FACHB-806 slowed down, while that of SCS-02 was not affected. The highest biomass concentrations of CCALA-415, SCS-02 and FACHB-806 were 4.84 g L^−1^ (day 12), 6.43 g L^−1^ (day 14) and 5.60 g L^−1^ (day 14), respectively. During the culture period, the color of CCALA-415, SCS-02 and FACHB-806 in the photobioreactors changed from light red (day 0) to dark red (day 8) and then to reddish brown (day 14) (Figure 2b). CCALA-415 had the fastest color change from light red to dark red, followed by SCS-02 and FACHB-806.

### 3.3. Phycobiliproteins Content

**Figure 3:**
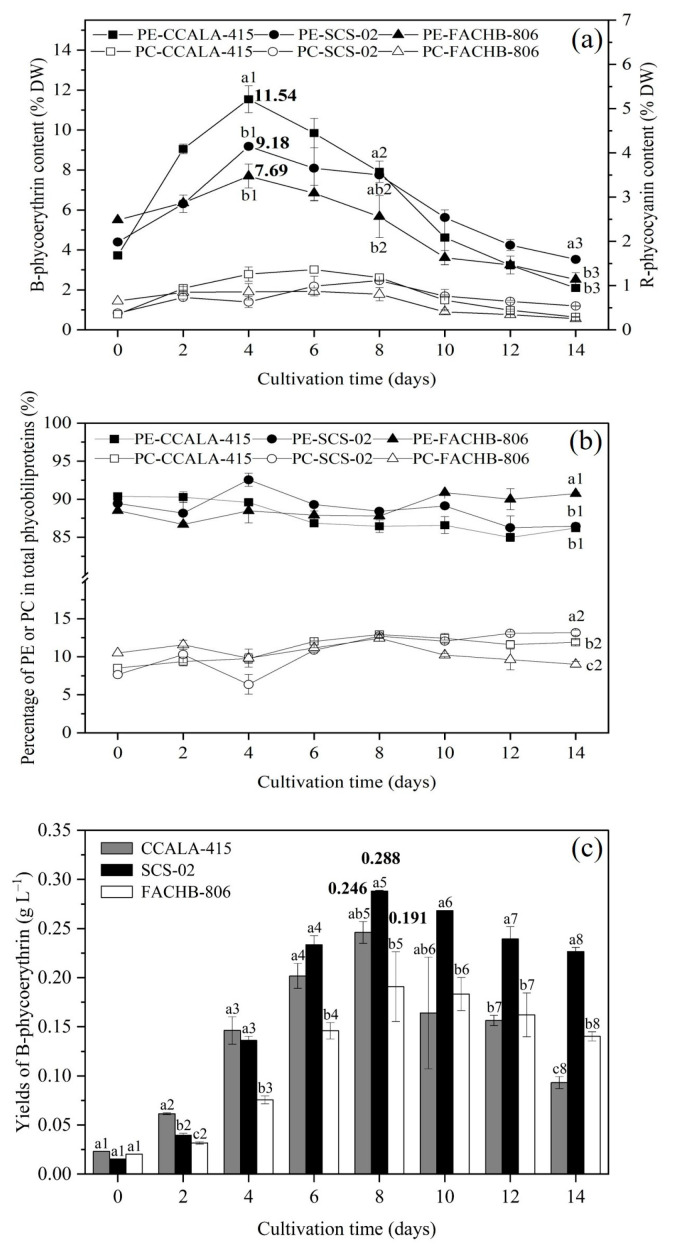
Accumulation characteristics of phycoerythrin and phycocyanin in *Porphyridium* *cruentum* CCALA-415, *Porphyridium* *purpureum* SCS-02 and *Porphyridium* *purpureum* FACHB-806. (**a**) The changes of B-phycoerythrin and R-phycocyanin content as a function of time; (**b**) The percentage of phycoerythrin and phycocyanin in total phycobiliproteins; (**c**) The yields of B-phycoerythrin as a function of time. PE: phycoerythrin; PC: phycocyanin. The values shown are the averages of two biological replicates and three technical replicates ± standard deviation. Different letters in Figure 3a denote significant differences among the B-phycoerythrin content in the three strains of *Porphyridium* at the same cultivation time (a1–c1: day 4; a2–c2: day 8; a3–c3: day 14). Different letters in Figure 3b denote significant differences among the percentage of B-phycoerythrin (a1–c1: day 14) or phycocyanin (a2–c2: day 14) in total phycobiliproteins in the three strains of *Porphyridium* at the same cultivation time. Different letters in Figure 3c denote significant differences among the yields of B-phycoerythrin in the three strains of *Porphyridium* at the same cultivation time (a1–c1: day 0; a2–c2: day 2; a3–c3: day 4; a4–c4: day 6; a5–c5: day 8; a6–c6: day 10; a7–c7: day 12; a8–c8: day 14) (level of significance, *p* < 0.05).

The phycoerythrin contents of CCALA-415, SCS-02 and FACHB-806 increased from day 0 to day 4 and decreased from day 4 to day 14 (Figure 3a). The maximum contents of phycoerythrin during the culture period for the three strains were 11.54% DW, 9.18% DW and 7.69% DW, respectively. On the end of the culture, the phycoerythrin contents of CCALA-415, SCS-02 and FACHB-806 decreased by 81.85%, 61.56% and 67.22%, respectively. Additionally, the changes in phycocyanin contents of CCALA-415, SCS-02 and FACHB-806 showed a similar trend with phycoerythrin contents. And the maximum contents during the culture period were 1.36% DW (day 6), 1.11% DW (day 8) and 0.87% DW (day 6), respectively. Compared to their maximum contents, the phycocyanin contents decreased by 78.76%, 51.81% and 71.07% on day 14, respectively.

The percentage of phycoerythrin in phycobiliprotein for CCALA-415, SCS-02 and FACHB-806 were 84.97–90.37% (avg. 87.66%), 86.26–92.55% (avg. 88.71%) and 86.69−90.86% (avg. 88.87%), respectively (Figure 3b). The percentage of phycocyanin in phycobiliprotein for CCALA-415, SCS-02 and FACHB-806 were 8.50–12.91% (avg. 11.06%), 6.36–13.16% (avg. 10.77%) and 9.01–12.43% (avg. 10.54%), respectively. The content of allophycocyanin in the three strains were very low, accounting for only 0.5−1.2% in phycobiliprotein.

On day 8, the phycoerythrin yields of CCALA-415, SCS-02 and FACHB-806 reached the highest levels (Figure 3c). The phycoerythrin yield of SCS-02 was 0.288 g L^−1^, which were 15% and 34% higher than that of CCALA-415 and FACHB-806, respectively. From day 8 to day 14, the yields of phycoerythrin of CCALA-415, SCS-02 and FACHB-806 decreased by 62%, 21% and 27%, respectively. Therefore, the yield of phycoerythrin of SCS-02 was significantly higher than that of CCALA-415 and FACHB-806 (*p* < 0.01) on day 14.

### 3.4. Amino acid Sequences of Phycoerythrin

**Figure 4:**
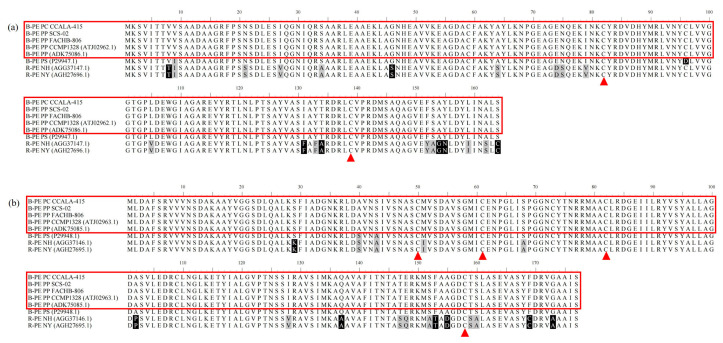
Amino acid sequence alignment of α subunit (**a**) and β subunit (**b**) of phycoerythrin from CCALA-415, SCS-02 and FACHB-806. PP: *Porphyridium purpureum*; PC: *Porphyridium cruentum*; PS: *Porphyridium sordidum*; NH: *Neopyropia haitanensis*; NY: *Neopyropia yezoensis*. Black shading indicates completely different amino acids; gray shading indicates similar amino acids; the label ▲ is the position where the subunit is attached to the chromophore.

The α subunits of phycoerythrin in CCALA-415, SCS-02 and FACHB-806 consisted of 495 bases, which encoded 164 amino acids (Figure 4a). The amino acid sequence of α subunits exhibited 100% sequence homology in the three strains and also 100% sequence homology with other two *P. purpureum* (ATJ02962.1 and ADK75086.1) recorded in the NCBI database. Compared to the amino acid sequence of α subunit in *Porphyridium sordidum*, *N. haitanensis* and *N. yezoensis*, the sequence identities were 99.78%, 87.73% and 87.73%, and the sequence positives were 99.39%, 96.32% and 96.32%, respectively.

The phycoerythrin β subunits of CCALA-415, SCS-02 and FACHB-806 consisted of 534 bases, which encoded 177 amino acids and also have 100% sequence homology (Figure 4b). In addition, the alignment with the phycoerythrin β subunit amino acid sequences (ATJ02963.1 and ADK75085.1) of two strains of *Porphyridium* in the NCBI database also reached 100% sequence homology. In comparison with the phycoerythrin β subunit of *P. sordidum*, *N. haitanensis* and *N. yezoensis*, the sequence identities were 98.87%, 90.40% and 90.40%, respectively, and the sequence positives were 100.00%, 96.05% and 96.05%, respectively.

### 3.5. Fluorescence Characteristics of Phycoerythrin

**Figure 5:**
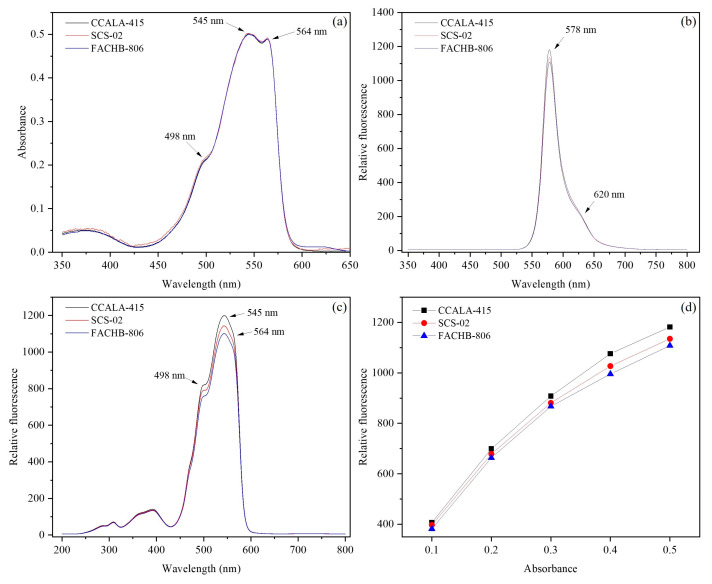
The fluorescence characteristics of phycoerythrin from *Porphyridium cruentum* CCALA-415, *Porphyridium purpureum* SCS-02 and *Porphyridium purpureum* FACHB-806. (**a**) The absorption spectra of the purified phycoerythrin at room temperature; (**b**) The fluorescence emission spectra of the purified phycoerythrin (the excitation wavelength at 545 nm); (**c**) The fluorescence excitation spectra of the purified phycoerythrin (the emission wavelength at 578 nm); (**d**) The relative fluorescence intensity at the emission wavelength of 578 nm (the excitation wavelength at 545 nm) under the OD_545_ of the purified phycoerythrin at 0.1, 0.2, 0.3, 0.4 and 0.5, respectively. OD_545_ represents the optical density at 545 nm. The values shown are the averages of three technical replicates ± standard deviation.

The purity (OD_545_/OD_280_) of the purified phycoerythrin in CCALA-415, SCS-02 and FACHB-806 were 9.2, 8.3 and 5.0, respectively. In the absorption spectra of phycoerythrin at wavelength of 350–800 nm (Figure 5a), three strains had no significant difference in the wavelength of the absorption peaks, 545 nm and 564 nm for the main absorption peaks and 498 nm for a shoulder peak.

In Figure 5b, CCALA-415, SCS-02 and FACHB-806 had similar fluorescence emission spectrum, with the main fluorescence peak at 578 nm and the shoulder peak at 620 nm. Meanwhile, CCALA-415 had the maximum fluorescence intensity at 578 nm, followed by SCS-02 and FACHB-806. In Figure 5c, CCALA-415, SCS-02 and FACHB-806 had similar fluorescence excitation spectrum, with the main fluorescence peak at 545 nm and two shoulder peaks at 498 nm and 564 nm.

Under the same OD_545_ of the purified phycoerythrin, CCALA-415 had stronger fluorescence intensity compared to SCS-02 and FACHB-806 (Figure 5d). For example, when OD_545_ of the purified phycoerythrin from CCALA-415 was 0.50, the relative fluorescence intensity was 1182, which was 4.1% and 6.7% higher than that from SCS-02 and FACHB-806, respectively. With the OD_545_ of the purified phycoerythrin increasing from 0.1 to 0.5, their relative fluorescence intensities at the excitation wavelength of 545 nm showed an exponential increasing trend for CCALA-415, SCS-02, and FACHB-806, rather than a linear increasing trend (Table 2).

### 3.6. Transcriptome Analysis

Total clean reads were obtained: 42,274,636 for CCALA-415, 48,996,844 for SCS-02 and 42,821,236 for FACHB-806 (Table 3). Q_20_ of the three strains were above 97% and the percentage of ambiguous bases (N%) were less than 0.002, indicating that the sequencing data had high accuracy and confidence. Meanwhile, CCALA-415, SCS-02 and FACHB-806 had 92.52%, 95.00% and 90.52% of the sequences matched to the reference genome, respectively.

**Figure 6:**
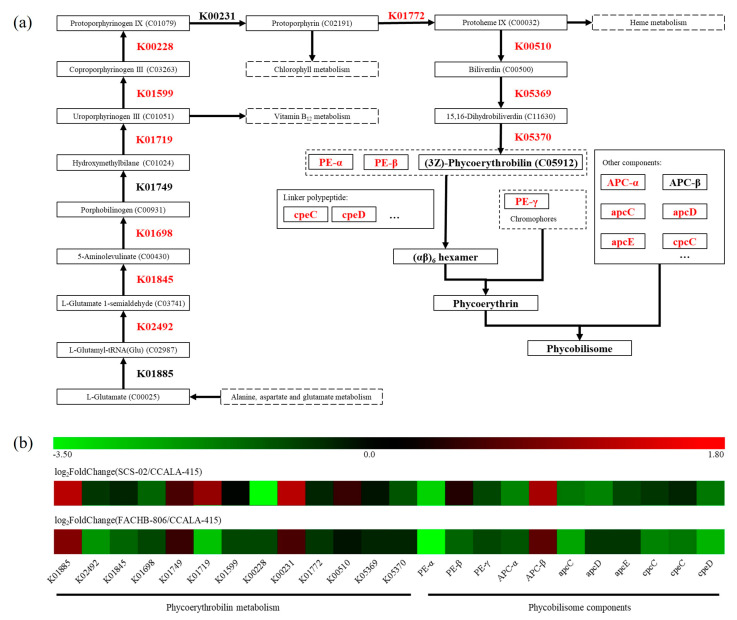
Distribution of differentially expressed genes in the phycoerythrin synthetic pathways of *Porphyridium*. (**a**) The metabolic pathways of phycoerythrin synthesis; (**b**) Heatmap of expression level from the activated and inhibited genes in K01885, K02492, K01845, K01698, K01749, K01719, K01599, K00228, K00231, K01772, K00510, K05369 and K05370 are KEGG entry. PE-α, PE-β and PE-γ are the α, β and γ subunits of phycoerythrin, respectively. APC-α and APC-β are the α and β subunits of allophycocyanin, respectively. ApcC, apcD, apcE, cpcC, cpeC and cpeD are phycobilisome linker proteins, respectively. *Porphyridium cruentum* CCALA-415 is the control. *Porphyridium purpureum* SCS-02 and *Porphyridium purpureum* FACHB-806 are the treatment.

A total of 24 genes related to the synthesis of phycoerythrobilin and phycobilisome were annotated (Table 4). As shown in Figure 6a, L-glutamate derived from the alanine, aspartate and glutamate metabolic pathways was converted to (3Z)-phycoerythrobilin through 13 steps reaction. Subsequently, (3Z)-phycoerythrobilin underwent ring-opening reaction and then attached to the α and β subunits of phycoerythrin. The α, β and γ subunits of phycoerythrin carrying chromophores were linked by linker proteins to form active phycoerythrin. As shown in Figure 6b, SCS-02 had 20 down-regulated genes and 4 up-regulated genes compared to CCALA-415. According to the value of log_2_FoldChange, the genes, including K02492, K01719, PE-α, apcC and cpeD of CCALA-415, showed the higher expressional level than that of SCS-02. There were 17 down-regulated genes and 7 up-regulated genes found in FACHB-806. The genes with the value of log_2_FoldChange greater than two included K00228 and PE-α. In summary, the phycoerythrin synthesis-related genes of CCALA-415 were generally up-regulated compared to those of SCS-02 and FACHB-806.

## 4. Discussion

High biomass concentration is the prerequisite for the high-yield phycoerythrin. In the present study, *P. purpureum* SCS-02 could maintain a rapid growth rate during the culture period. The highest biomass concentration of SCS-02 reached 6.43 g L^−1^, which was 19% higher than that of CCALA-415 and FACHB-806. This was a high biomass concentration with the previous study [34]. Most previous studies showed that *Porphyridium* could simultaneously accumulate biomass and high content of phycoerythrin in the favorable culture medium. Su et al. (2016) found that the ASW medium contributed to higher biomass production of *Porphyridium* than that in the other three media, including KOCK medium, Pringsheim medium II and F/2 medium [35]. Medina-Cabrera et al. (2020) reported that *P. purpureum* SAG 1380-1a grew well in the modified ASW medium [36]. Li et al. (2020) showed that ASW medium was the better for the growth of *P. purpureum* CCMP 1328 compared to F/2 and KOCK medium [20]. Thus, the modified ASW medium was used in the present study. The color of the culture changed significantly with increasing culture time. The color change might be mainly related to the content of phycoerythrin.

Low light could enhance the synthesis of phycobiliproteins in *Porphyridium* [37]. As cell density increased, light energy for individual cell decreased. Theoretically, the algal cells could synthesize more phycobiliproteins to improve the absorption of light energy. However, the phycoerythrin content of the three strains started to decrease after reaching the highest content on day 4. The results were similar with the report from Li et al. (2020). The reduction in phycoerythrin content was notable at low sodium nitrate concentration (0.25 g L^−1^) compared to that at high sodium nitrate concentration (1.00 g L^−1^), speculating that the carbon to nitrogen ratio might be the key factor for the decrease in phycoerythrin content [20]. Notably, SCS-02 showed the least decrease in the contents of phycoerythrin and phycocyanin. It might be less sensitive to environmental changes.

Phycoerythrin is mainly located at the periphery of the disc-like structure of phycobilisome and assembles with phycocyanin and allophycocyanin in a certain proportion to form phycobilisome [38]. For the three strains, the percentage of phycobiliproteins (including phycoerythrin, phycocyanin and allophycocyanin) were relatively stable during the culture period. The average percentage of phycoerythrin in total phycobiliproteins was 88%. Moreover, only a small amount of phycocyanin and allophycocyanin were detected. The results were consistent with *P. purpureum* IBSS-70 [19]. The phycoerythrin, phycocyanin and allophycocyanin in *P. purpureum* IBSS-70 accounted for approximately 85%, 12% and 3% in the total phycobiliproteins, respectively [19]. In fact, the percentage of phycoerythrin in phycobiliprotein of *Porphyridium* was remarkably high among red algae. Lee et al. (2021) reported that the percentage of phycoerythrin in a red macroalga *Colaconema* sp. reached 65% of phycobiliproteins [39]. Among the phycobiliproteins of *N. yezoensis*, the percentage of phycoerythrin accounted for about 40% [13]. Currently, commercial phycoerythrin was mainly extracted from the red macroalga *Neopyropia* [40], such as *N. linearis* [41] and *N. dioica* [42], which contained 0.29% DW and 0.97% DW of phycoerythrin, respectively. However, the phycoerythrin content of the three strains of *Porphyridium* reached an average of 6% DW, which was higher than that of *Neopyropia*. Moreover, the phycoerythrin content of *Porphyridium* was remarkably higher than that of 15 strains of Arctic red macroalgae (0.30–3.45% DW) [43]. The yield of phycoerythrin was the product of the biomass concentration and the content of phycoerythrin. In the present study, the yield of phycoerythrin of the three strains ranged from 0.191 to 0.288 g L^−1^, which were at a high level in the previous studies related to *Porphyridium* [18,20,44]. In addition, SCS-02 showed the least decrease in the yield of phycoerythrin on day 8 to day 14, indicating that SCS-02 could retain more phycoerythrin in response to the nutrition depletion. Overall, SCS-02 was a suitable strain for phycoerythrin production.

The amino acid sequences of α and β subunit in CCALA-415, SCS-02 and FACHB-806 were 100% sequence homology. It was indicated that *P. purpureum* and *P. cruentum* might be no different in the amino acid sequences of α and β subunits. In addition to the two species mentioned above, *Porphyridium aerugineum* and *Porphyridium sordidum* were also widely studied, both of which were green under the light microscope [36,45]. However, *P. sordidum* contained 29% phycoerythrin [46], while *P. aerugineum* did not contain optically active phycoerythrin [47]. The amino acid sequences of the α subunits (P29947.1) and β subunits (P29948.1) of *P. sordidum* were 99.39% and 100%, similar to that of the α and β subunits of the three strains in our study. Moreover, only at position 96α were the sequences substituted by a difference amino acid (neutral amino acid cysteine was replaced by the acidic amino acid aspartic acid). The α and β subunits of B-phycoerythrin were linked to the chromophore through cysteines at positions of 82α, 139α, 50β, 61β, 82β and 158β [32]. Compared with the phycoerythrin α and β subunits of *N. haitanensis* and *N. yezoensis*, the positives of the amino acid sequence between *Porphyridium* and *N. haitanensis* as well as *Porphyridium* and *N. yezoensis* could reach 96.32% and 96.05%, and there was no difference in the above-mentioned six sites linked to the chromophore. Therefore, it was speculated that the α and β subunits of phycoerythrin were highly conserved, and the differences in α and β subunits amino acid sequence of phycoerythrin was not the main factor affecting the content of phycoerythrin.

There are two types of phycoerythrin, B-phycoerythrin and b-phycoerythrin [48,49]. B-phycoerythrin consisted of α, β, and γ subunits. The wavelength of the main absorption peaks for B-phycoerythrin were 545 nm and 565 nm. The chromophore carried by γ subunits contributed to the shoulder peak at 498 nm. In contrast, because the γ subunit was not present, only two absorption peaks at 545 nm and 556 nm were detected [50]. Due to the presence of absorption peaks at 498 nm, 545 nm and 565 nm, it was conducted that the type of phycoerythrin in CCALA-415, SCS-02 and FACHB-806 were B-phycoerythrin. The fluorescence emission (Figure 5b) and fluorescence excitation spectra (Figure 5c) of the purified phycoerythrin of three strains of *Porphyridium* were similar, but the maximum relative fluorescence intensities were different, ranging from high to low was CCALA-415, SCS-02 and FACHB-806. The result might be related to the purity (OD_545_/OD_280_) of phycoerythrin, which was 9.2, 8.3 and 5.0 for CCALA-415, SCS-02 and FACHB-806, respectively. The fluorescence characteristics of phycoerythrin were dependent on the internal attached chromophore and the environmental conditions. The histidine conformation at position 88α of phycoerythrin altered when the pH of the buffer changed from 5 to 8, resulting in an obvious change in the absorption spectrum [32]. The relative fluorescence intensity decreased exponentially with the decreasing phycoerythrin concentration, which was inconsistent with that reported by Lee et al. (2021) [4]. The purified phycoerythrin solution (the purity was 4.3) of *Colaconema* sp. were diluted 2−256 times. The relative fluorescence intensity decreased in a linear trend with the increasing dilution multiple [4]. In our study, the relative fluorescence intensity was lower than the expectation as the concentration of phycoerythrin decreased, presumably for the following reason. In addition to the common α_6_β_6_γ protein complex in B-phycoerythrin solution (pH 7), other subunit combination forms such as α_3_β_3_, α_12_β_12_γ_2_ and αβ, were also presented [51]. The depolymerization of subunits possibly occurred due to changes in the concentration of phycoerythrin, resulting in the decrease in relative fluorescence intensity. In summary, there was no significant difference in the fluorescence characteristics of CCALA-415, SCS-02 and FACHB-806.

B-phycoerythrin hexamer (αβ)_6_γ contains a total of 34 chromophores, including 32 phycoerythrobilin and 2 phycourobilin. Thus, phycoerythrobilin is the main chromophore of B-phycoerythrin. In order to explain the differences in phycoerythrin content among the three strains of *Porphyridium*, the gene expression levels of the key enzymes in phycoerythrin synthesis pathway were investigated by transcriptome. The synthesis pathways of phycoerythrobilin, chlorophyll and heme shared an important precursor, 5-aminolevulinate (5-ALA). 5-ALA was synthesized in plants through the C_5_ pathway [52], which was composed from L-glutamate via glutamyl-tRNA synthetase (K01885), glutamyl-tRNA reductase (K02492) and glutamate-1-semialdehyde 2,1-aminomutase (K01845). The gene expression levels of K02492 and K01845 in CCALA-415 were higher than that of FACHB-806 and SCS-02. Meanwhile, the gene expression level of porphobilinogen synthase (K01698), which catalyzed 5-ALA to porphobilinogen, was also up-regulated in CCALA-415. This suggests that the synthesis of 5-ALA were enhanced in CCALA-415. In the last step of phycoerythrobilin synthesis, biliverdin was converted to (3Z)-phycoerythrobilin by 15,16-dihydrobiliverdin: ferredoxin oxidoreductase (K05369) and phycoerythrobilin: ferredoxin oxidoreductase (K05370). For CCALA-415, the gene expression levels of K05369 and K05370 were up-regulated compared to SCS-02 and FACHB-806. Therefore, it was speculated that more amounts of phycoerythrobilin were synthesized in CCALA-415 than in SCS-02 and FACHB-806.

The synthesis of α and β subunits were adjusted by the key genes *peA* and *peB*, respectively [53]. Huang et al. (2017) reported that the expression of *peA* and *peB* in a green mutant of red macroalga *Gracilariopsis lemaneiformis* was significantly down-regulated by 1.2-fold compared to the wild type [54]. In our study, the gene expression level of the α subunit and two phycoerythrin-associated linker peptides (cpeC and cpeD) in CCALA-415 was up-regulated compared to that of FACHB-806 and SCS-02. For the other components of phycobilisome, the gene expression levels of allophycocyanin α subunit, allophycocyanin-associated linker proteins (apcC, apcD and apcE) and phycocyanin-associated linker protein (cpcC) were up-regulated in CCALA-415. It was hypothesized that CCALA-415 was active in the whole phycoerythrin synthesis process and that more phycoerythrin was synthesized compared to FACHB-806 and SCS-02. At the same time, transcriptome data were consistent with the results on the phycoerythrin content of the three strains (Figure 3b). The phycoerythrin content of CCALA-415 was higher than that of SCS-02 and FACHB-806. However, SCS-02 was still considered as the most promising strain for phycoerythrin production among the three stains of *Porphyridium* due to its high yield of phycoerythrin.

## 5. Conclusions

*P. cruentum* CCALA-415, *P. purpureum* SCS-02 and *P. purpureum* FACHB-806 had high sequence homology on 18s rDNA and ITS. There were no significant differences in the amino acid sequences of the phycoerythrin α and β subunits and their fluorescence characteristics. Among the key enzymes related to phycoerythrin synthesis, porphobilinogen synthase and oxygen-dependent coproporphyrinogen-III oxidase in phycoerythrobilin synthesis had a significant effect on the content of phycoerythrin. Among the three strains, *P. purpureum* SCS-02 had the highest yield of phycoerythrin, which was more suitable for phycoerythrin production.

## Figures and Tables

**Table 1:** Primers for gene sequence amplification.

Gene Name	Primer	Primer Sequence
18s rDNA	18s-F	5′-CTGGTTGATCCTGCCAGT-3′
	18s-R	5′-CACCTACGCAAACCTTGTTACGACTT-3′
ITS	ITS-F	5′-GGAAGGAGAAGTCGTAACAAGG-3′
	ITS-R	5′-TCCTCCGCTTATTGATATGC-3′
α subunit of phycoerythrin	AS	5′-ATGAAATCAGTTATTACTACTGTTGTAAGTGCAGCTG-3′
	AAS	5′-CTATGAAAGTGCGTTAATTAAGTAG-3
β subunit of phycoerythrin	BS	5′-ATGCTTGACGCATTTTCTAGAGT-3′
	BAS	5′-TTAGCTAATTGCTGCACCAA-3′

**Table 2:** The fitted curves of relative fluorescence intensity at the emission wavelength of 578 nm (the excitation wavelength at 545 nm) for *Porphyridium* *cruentum* CCALA-415, *Porphyridium* *purpureum* SCS-02 and *Porphyridium* *purpureum* FACHB-806.

Strain	Fitted Curves	R^2^
CCALA-415	y = 486.33ln(x) + 1508.6	0.9960
SCS-02	y = 461.16ln(x) + 1444.0	0.9972
FACHB-806	y = 452.21ln(x) + 1411.4	0.9980

**Table 3:** Summary of output statistics by Illumina 2000 sequencing for three stains of *Porphyridium*.

Samples	Total Raw Reads	Total Clean Reads	Total Clean Nucleotides (nt)	Q_20_ (%)	N (%)	Map to Reference Genome (%)
CCALA-415	47,261,608	42,274,636	6341,195,400	97.23	0.001258	92.86%
SCS-02	53,769,454	48,996,844	7349,526,600	97.42	0.001235	95.06%
FACHB-806	47,496,606	42,821,236	6423,185,400	97.51	0.001244	90.65%

**Table 4:** The differentially expressed genes in the phycobiliproteins metabolism pathway.

	KEGG Entry	Protein	Abbreviation	SCS-02 vs. CCALA-415	FACHB-806 vs. CCALA-415
Phycoerythrobilin metabolism	K01885	Glutamyl-tRNA synthetase	-	Up	Up
	K02492	Glutamyl-tRNA reductase	-	Down	Down
	K01845	Glutamate-1-semialdehyde-2,1-aminomutase	-	Down	Down
	K01698	Porphobilinogen synthase	-	Down	Down
	K01749	Porphobilinogen deaminase, chloroplastic	-	Up	Up
	K01719	Uroporphyrinogen-III synthase	-	Down	Up
	K01599	Uroporphyrinogen decarboxylase	-	Down	Down
	K00228	Oxygen-dependent coproporphyrinogen-III oxidase	-	Down	Down
	K00231	Protoporphyrinogen oxidase	-	Up	Up
	K01772	Protoporphyrin/coproporphyrin ferrochelatase	-	Down	Down
	K00510	Heme oxygenase 1	-	Down	Up
	K05369	15,16-dihydrobiliverdin: ferredoxin oxidoreductase	-	Down	Down
	K05370	Phycoerythrobilin: ferredoxin oxidoreductase	-	Down	Down
Phycobilisome components	K05376	Phycoerythrin alpha subunit	PE-α	Down	Down
	K05377	Phycoerythrin beta subunit	PE-β	Down	Up
	K05378	Phycoerythrin gamma chain	PE-γ	Down	Down
	K02092	Allophycocyanin alpha subunit	APC-α	Down	Down
	K02093	Allophycocyanin beta subunit	APC-β	Up	Up
	K02094	Phycobilisome 7.8 kDa linker polypeptide, allophycocyanin-associated, core	apcC	Down	Down
	K02095	Phycobilisome linker polypeptide	apcD	Down	Down
	K02096	Phycobilisome linker polypeptide	apcE	Down	Down
	K02286	Phycobilisome 32.1 kDa linker polypeptide, phycocyanin-associated, rod	cpcC	Down	Down
	K05378	Phycobilisome 31.8 kDa linker polypeptide, phycoerythrin-associated, rod	cpeC	Down	Down
	K05379	Phycobilisome 27.9 kDa linker polypeptide, phycoerythrin-associated, rod	cpeD	Down	Down

Up: the expression of gene is up-regulated. Down: the expression of gene is down-regulated. *Porphyridium cruentum* CCALA-415 is the control. *Porphyridium purpureum* SCS-02 and *Porphyridium purpureum* FACHB-806 are treatment group.

## Data Availability

Data is contained within the article.
